# Use of Some Asteraceae Plants for the Treatment of Wounds: From Ethnopharmacological Studies to Scientific Evidences

**DOI:** 10.3389/fphar.2018.00784

**Published:** 2018-08-21

**Authors:** Alexsander R. Carvalho, Roseana M. Diniz, Mariela A. M. Suarez, Cristiane S. S. e S. Figueiredo, Adrielle Zagmignan, Marcos A. G. Grisotto, Elizabeth S. Fernandes, Luís C. N. da Silva

**Affiliations:** Programa de Pós-Graduação, Universidade Ceuma, São Luís, Brazil

**Keywords:** *Ageratina pichinchensis*, *Calendula officinalis*, silibinin, jaceosidin, drug development, ethnomedicine

## Abstract

Severe wounds result in large lesions and/or loss of function of the affected areas. The treatment of wounds has challenged health professionals due to its complexity, especially in patients with chronic diseases (such as diabetes), and the presence of pathogens such as *Staphylococcus aureus* and *Pseudomonas aeruginosa*. Taking this into consideration, the development of new therapies for wound healing requires immediate attention. Ethnopharmacological studies performed in different countries have shown the use of several plants from the Asteraceae family as wound-healing agents. Evidences gained from the traditional medicine have opened new ways for the development of novel and more efficient therapies based on the pharmacological properties of these plants. In this article, we discuss the literature data on the use of Asteraceae plants for the treatment of wounds, based on the ethnopharmacological relevance of each plant. Special attention was given to studies showing the mechanisms of action of Asteraceae-derived compounds and clinical trials. *Ageratina pichinchensis* (Kunth) R.M. King and H. Rob. and *Calendula officinalis* L. preparations/compounds were found to show good efficacy when assessed in clinical trials of complicated wounds, including venous leg ulcers and foot ulcers of diabetic patients. The compounds silibinin [from *Silybum marianum* (L.) Gaertn.] and jaceosidin (from *Artemisia princeps* Pamp.) were identified as promising compounds for the treatment of wounds. Overall, we suggest that Asteraceae plants represent important sources of compounds that may act as new and efficient healing products.

## Introduction

Wounds, especially of chronic nature, cause a serious public health concern as they negatively affect the quality of life of a large number of people, showing psychological, social, and economic impacts (Vowden and Vowden, 2016; Guest et al., 2017). When not properly treated, their associated lesions can become larger and result in the loss of function of the affected areas. Wounds are classified on the basis of the various factors such as location, borders, size, tissue type, secretion, odor, edema, and pain (Frykberg and Banks, 2015; Wernick and Stawicki, 2018). Based on these characteristics, they can be simple or complex, deep or superficial, acute or chronic, sterile or contaminated, or even defined by the type of healing (Morton and Phillips, 2016; Karthik et al., 2018).

Based on its complexity, a wound is considered as simple when it is able to spontaneously evolve to resolution or as complex when lesions are extensive and/or deep and require special resources or more specialized treatment for its complete healing (Wernick and Stawicki, 2018). Based on its depth, a wound is superficial when it is restricted to the epidermis or dermis or deep when it affects the subcutaneous tissue, muscles, and/or bones (Shin et al., 2017; Podd, 2018). Wounds can be also classified as acute or chronic, with the former achieving resolution within 3 weeks and minimal or no scar tissue formation, whereas the latter may take several weeks to heal and can often lead to the loss of function of the affected tissues (Frykberg and Banks, 2015; Jørgensen et al., 2016). The healing time is influenced by different factors such as the presence of comorbidities (diabetes, hypertension, neurological lesions, among others), infection, aging, nutritional status, personal care, and appropriate and timely treatment (Manrique et al., 2015; Kulprachakarn et al., 2017; Hou and Kim, 2018; Long et al., 2018; Yuan et al., 2018).

The treatment of chronic wounds is complex and can include the administration of vascular endothelial growth factor or erythropoietin, which may not always be efficient and present high costs and has short half-life and side effects (Hong and Park, 2014; Arslantas et al., 2015; Yu et al., 2018). Moreover, the treatment of chronic wounds often requires the use of antimicrobials due to their multifactorial nature (Karthik et al., 2018). Considering this aspect, the development of new therapies for wound healing requires immediate attention. Plant-derived products have presented protective actions in wound care, and the healing activity of several active compounds has been shown (Agar et al., 2015; Neamsuvan and Bunmee, 2016; Jaric et al., 2018). Ethnopharmacological studies performed in different countries have shown that many plants from the Asteraceae family may be useful as sources of healing agents and therefore aid in the treatment of different types of wounds. As such, their pharmacological potential has been explored in an attempt to develop novel and more efficient therapies to accelerate healing and diminish the loss of function of tissues in the wounded area (Agar et al., 2015; Neamsuvan and Bunmee, 2016; Jaric et al., 2018). This article discusses the scientific evidences supporting the use of Asteraceae plants and their derived compounds as healing therapies. The main focus was given to plants with ethnopharmacological relevance, especially to the mechanism of action of their isolated compounds and clinical trials assessing their efficacy.

## Overview of Wound Healing

**FIGURE 1:**
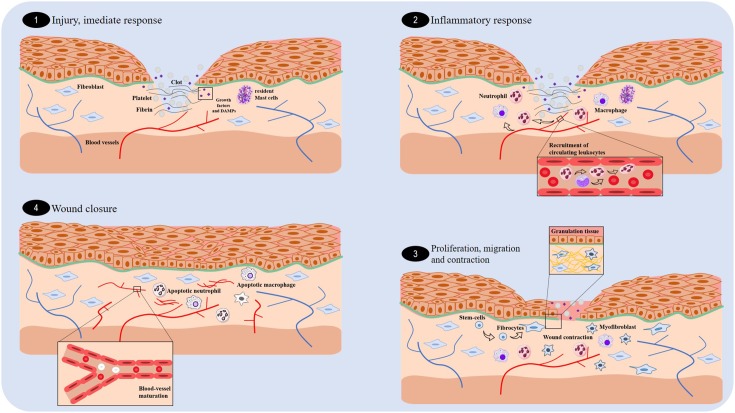
Overview of the essential stages of wound healing. There are four fundamental stages involved in wound healing: the immediate response **(1)**, inflammation **(2)**, proliferation **(3)**, and wound closure **(4)**.

Wound healing consists of a coordinated cascade of cellular and biochemical events that interact with the tissue reconstitution (Eming et al., 2014; Lee and Jang, 2018; Nagle and Wilbraham, 2018). This process consists of three distinct and superposed phases: inflammation, proliferation, and tissue remodeling (**Figure 1**) (Eming et al., 2014). Inflammation occurs soon after lesion. At this stage, a blood clot is formed to cease bleeding and also to make a viable matrix rich in growth factors and chemokines, which in turn contribute to the migration of leukocytes and stromal cells (Rousselle et al., 2018). After 24 h, neutrophils appear at the margins of the wounds, and the process of sterilization and waste degradation begin. This first stage of the healing process is normally completed within 3 days following surgical or acute wounds (Gurtner et al., 2008; Shaw and Martin, 2009).

Proliferation is the phase responsible for the wound closure, occurring 4 days following a wound. It involves re-epithelization (movement of epithelial cells), formation of granulation tissue (responsible for filling the injured tissue), and angiogenesis (Kanji and Das, 2017; Zomer and Trentin, 2018). Fibroblasts produce the new extracellular matrix necessary for the cell growth, whereas the new blood vessels carry oxygen and the nutrients necessary for the local cellular metabolism (Shaw and Martin, 2009; Rousselle et al., 2018). Two weeks after the lesion, there is a vasculature regression, and the granulation tissue is converted into an avascular scar without inflammation, which is covered by intact epithelium, as a result of collagen deposition (Broughton et al., 2006; Eming et al., 2014). Scar contraction occurs in large lesions due to the activity of myofibroblasts—fibroblast-like cells that have the contractile ability of smooth muscle cells (Wernick and Stawicki, 2018; Zomer and Trentin, 2018).

Finally, the remodeling phase begins approximately 3 weeks after the lesion. This phase is characterized by a random deposition of collagen, and then, metalloproteinases (produced by macrophages, neutrophils, fibroblasts, and epithelial cells) regulate the degradation and deposition of extracellular matrix, which are essential for wound re-epithelization (Gurtner et al., 2008; Caley et al., 2015; Kanji and Das, 2017; Rousselle et al., 2018; Zomer and Trentin, 2018). Consequently, larger collagen fibers are formed and organized according to the direction of the adjacent connective tissue. At the end of this stage, there is a limited regeneration of the skin attachments, such as hair follicles and glands, and a pale-colored scar with up to 80% of the original tensile strength present (Broughton et al., 2006; Gurtner et al., 2008; Eming et al., 2014).

## Plants From Asteraceae Family as Wound-Healing Agents

Plants have been used for medicinal purposes for many years as shown in previous studies (Abd Rani et al., 2018; Ricardo et al., 2018; Tiwari et al., 2018). In this context, plants from the Asteraceae family are well known for their ethnopharmacological importance for many communities (Rodriguez-Chavez et al., 2017; Tewari et al., 2017; Saleh and Van Staden, 2018), and this family is widely distributed and is considered to be the largest family of flowering plants in the world (Gao et al., 2010). Due to their distribution and ethnopharmacological importance, several plant-derived products from this family have been studied, with some of their pharmacological activities already identified. These include anti-inflammatory (George Kallivalappil and Kuttan, 2017), antimicrobial (Ghaderi and Sonboli, 2018), antioxidant (Babota et al., 2018), anti-protozoa (Garcia et al., 2017), and healing activities (Ozbilgin et al., 2018). Some species [such as *Calendula officinalis* L., *Achillea millefolium* L.*, Neurolaena lobata* (L.) R.Br. ex Cass.] have been specially described in the literature due to their therapeutic potential for the treatment of wounds (Parente et al., 2012; Nayak et al., 2014). Their efficacy has been suggested to be related to their ability to promote the proliferation of keratinocytes and thus the remodeling of the extracellular matrix (Speroni et al., 2002; Rosa Ados et al., 2014). Hence, the therapeutic use of these plants will now be discussed. The most relevant studies (i.e., those that provide insights into the mechanism of action) are summarized in **Table 1**.

**Table 1:** Use of some Asteraceae plants for the treatment of wounds *in vivo* and *in vitro.*

Species	Popular name	Product	Type of study	Conclusions	Reference
*Blumea balsamifera* (L.)	Sambong	Leaf extract	*In vivo* study with Sprague-Dawley rats	The extract induced wound contraction, capillary regeneration, collagen deposition, and re-epithelization	Pang et al., 2017
		Volatile oil	*In vivo* study with Kun- Ming mice	The topical application of the volatile oil promoted capillary regeneration, blood circulation, collagen deposition, granular tissue formation, epithelial deposition, and wound contraction	Pang et al., 2014b
		Silibinin-based gel	*In vivo* study with Swiss mice	The formulation induced the production of collagen fibers, fibroblasts, and proliferating blood capillaries (angiogenesis)	Samanta et al., 2016
*Acmella oleracea*	Jambu	Rhamnogalacturonan	*In vivo* study in Wistar Rats with gastric ulcers	The treatment reduced the gastric lesions due to its anti-inflammatory and antioxidant mechanisms It also induced cellular proliferation	Maria-Ferreira et al., 2014
*Achillea asiatica*		Ethanolic extract	*In vivo* study with Sprague-Dawley rats and *in vitro* study with Hs68 fibroblasts	The extract enhanced healing by promotion of keratinocyte differentiation and motility and anti-inflammatory effects. It induced the expression of β-catenin, collagen, and keratinocyte differentiation markers	Dorjsembe et al., 2017
*Artemisia princeps* Pampanini	Korean wormwood, Korean mugwort, and Japanese mugwort	Jaceosidin	*In vitro* study with HUVEC^1^	Jaceosidin promoted proliferation, migration, differentiation of human endothelia cells, and angiogenesis	Lee et al., 2014
*Calendula officinalis*	Pot marigold	Hydroalcoholic extract	*In vivo* study with BALB/c mice and *in vitro* study with HDF^2^	The extract was able to induce tissue granulation, proliferation, and cell migration	Dinda et al., 2016
		Tincture	*In vitro* study with HI-38^3^, NIH-3T3^4^, HeLa^5^, HDF^2^	The treatment potentiated wound healing by stimulating fibroblast proliferation and migration in a PI3K-dependent pathway	Dinda et al., 2015
		Oil	*In vivo* study in foot ulcers of diabetic patients	The use of low-intensity laser therapy associated with *C. officinalis* oil caused analgesic and reduced inflammation	Carvalho et al., 2016
*Achyrocline alata*	Jateí-ka-há	Extract	*In vivo* study with mice	The extract accelerated the healing by decreasing the initial inflammatory response and promoted re-epithelization and collagen remodeling	Pereira et al., 2017

*^1^HUVECs, human umbilical vascular endothelial cells; ^2^HDF, human primary dermal fibroblast cells; ^3^HI-38, human lung fibroblast cells; ^4^NIH-3T3, Swiss albino mouse fibroblast cells; ^5^HeLa, human cervical carcinoma cells.*

### *Blumea balsamifera* (L.) DC.

*Blumea balsamifera* (L.) DC. is a plant used in the traditional medicine of several Asiatic countries, where it is popularly known as Ainaxiang (De Boer and Cotingting, 2014; Ong and Kim, 2014; Sujarwo et al., 2015). Its leaves are rich in volatile compounds such as L-borneol (major compound), terpenoids, fatty acids, phenols, alcohols, aldehydes, ethers, ketones, pyridines, furans, and alkanes (Pang et al., 2014a), which may contribute to the healing properties of *B. balsamifera*. Indeed, the topical application of the volatile oil obtained from the leaves of *B. balsamifera* in wounded Kun-Ming mice enhanced angiogenesis and collagen deposition, and additionally induced epithelial deposition and formation of granular tissue. This effect on the proliferation phase of healing was suggested to be associated with the increased production of the neuropeptide substance P (Pang et al., 2014b). The volatile oil also accelerated the healing of Sprague-Dawley rats with burn injuries by triggering the release of growth factors in the tissue and decreasing the plasma concentrations of pro-inflammatory cytokines (TNFα and IL-1) (Fan et al., 2015).

Pang et al. (2017) in their study evaluated the healing actions of a flavonoid-rich leaf extract from *B. balsamifera* on skin wounds of Sprague-Dawley rats. This extract caused wound contraction, capillary regeneration, collagen deposition, and re-epithelization 7 days following treatment. These alterations were associated with the enhanced expression of vascular endothelial growth factor, transforming growth factor-β1, and CD68 antigen in rat wound tissues. Different compounds were detected in the extract including 16 flavonoid aglucons, 5 flavonoid glycosides, 5 chlorogenic acid analogs, and 1 coumarin (Pang et al., 2017).

### Silibinin From *Silybum marianum* (L.) Gaertn.

**FIGURE 2:**
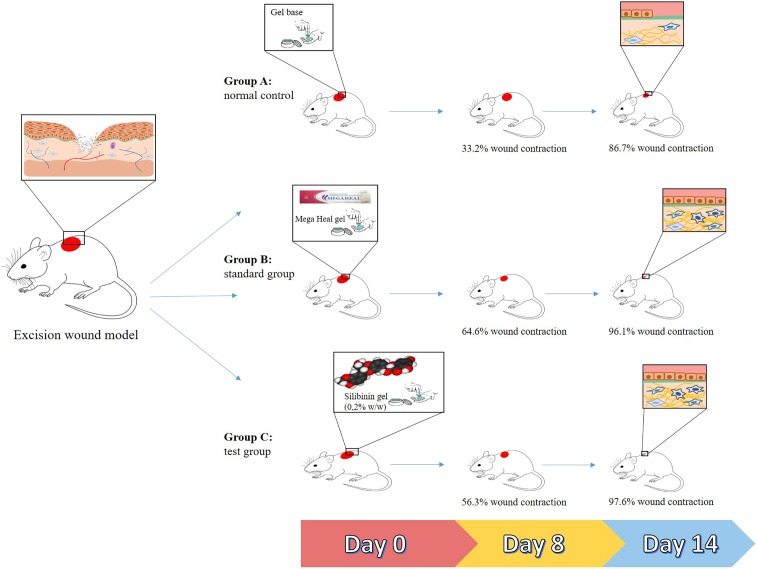
Summarized representation of the *in vivo* actions of silibinin (from *Silybum marianum*) in wound healing. Group A: mice treated with a gel base (controls). Group B: mice treated with a commercially available gel (Mega Heal gel; Aristo Pharmaceuticals Ltd.; positive control). Group C: mice treated with a silibinin-based gel (0.2% w/w). After 14 days of treatment, the mean sizes of the wounds from mice of groups B and C were smaller than those of group A.

*Silybum marianum* (L.) Gaertn. is another plant of ethnopharmacological importance in wound healing (Hudaib et al., 2008; Aziz et al., 2016). Evidences have shown that silymarin, an extract from its seeds, increases epithelization and decreases inflammation in albino rats subjected to the excision wound (Sharifi et al., 2012). It was also shown that this extract protects human fibroblasts from lipopolysaccharide (LPS)-induced oxidative stress (Sharifi et al., 2013). Similarly, the silymarin-derived compound silibinin (flavonoid) accelerated the closure of skin wounds in rats by upregulating the expression of stromelysin 1 hydroxyproline, glycosaminoglycans, and collagen (important constituents of extracellular matrix) (Tabandeh et al., 2013). This compound was also found to reduce the toxic effects caused by nitrogen mustard in the mouse skin (Balszuweit et al., 2013). This action was associated with an inhibition of oxidative stress and inflammation (Jain et al., 2015). As shown in another study, the repeated topical application (14 days treatment) of a silibinin-based gel resulted in an efficient wound healing strategy, by acting on tissue re-epithelization, collagen production, and deposition of granulation tissue (as shown in **Figure 2**) (Samanta et al., 2016).

### *Calendula officinalis* L.

*Calendula officinalis* L. (or calendula) is a species used in the treatment of wounds in Europe since 13th century, and a large number of cosmetic and personal care products have been developed using its compounds (Parente et al., 2012). Its use as a healing agent is supported by different *in vivo* and *in vitro* studies. In one of these studies, the ethanolic extract obtained from *C. officinalis* flowers, and its dichloromethane and hexanic fractions were found to increase angiogenesis in both chorioallantoic membranes (CAMs) of embryonated eggs and rat with skin wounds. This effect on vessels was related to discrete infiltration of inflammatory cells and increased the collagen deposition (Parente et al., 2011, 2012). Recently, a cream containing the glycolic extract from *C. officinalis* flowers was found to enhance collagen organization in the initial phase of the healing process, and this was correlated with an increase in the concentrations of hydroxyproline, an indicator of the collagen content in the tissue (Aro et al., 2015).

*In vitro* studies were performed to provide more insights into the mechanisms of action involved in the healing action of a product based on the hydroalcoholic extract of *C. officinalis* (approved by the European Medicines Agency (Nicolaus et al., 2017). *C. officinalis* tincture was able to increase the proliferation and the migration of fibroblasts in a PI3K-dependent pathway, with activation of FAK and Akt. Flavonol glycosides were the major compounds detected in this extract (Dinda et al., 2015). Human keratinocytes treated with *C. officinalis* flower extracts (*n*-hexanic and ethanolic extracts) exhibited the increased expression of IL-8 and activation of the transcription factor NF-κB, in addition to the enhanced migration ability. The ethanolic extract of this plant was also able to inhibit collagenase activity in human dermal fibroblasts. These effects were attributed to the presence of flavonoids and saponins in the extract (Nicolaus et al., 2017).

The hydroalcoholic extract and its aqueous fraction of *C. officinalis* (rich in rutin and quercetin-3-*O*-glucoside) exhibited significant *in vitro* effects on the proliferation and migration of human dermal fibroblasts, in addition to the increased expression of connective tissue growth factor and α-smooth muscle actin, proteins that favor healing by activating cell proliferation, migration, adhesion, and tissue repair. The topical application of hydroalcoholic extract or its aqueous fraction of *C. officinalis* on excisional wounds of BALB/c mice accelerated wound contraction by increasing the tissue levels of connective tissue growth factor and α-smooth muscle actin (Dinda et al., 2016).

Particularly noteworthy was the commercially available product containing the hydroglycolic extract of *C. officinalis* (Plenusdermax) as it promoted wound epithelization, thus decreasing the healing time in patients with venous leg ulcers (Buzzi et al., 2016). Another study showed that the use of low-intensity laser therapy associated with *C. officinalis* oil causes analgesia, in addition to the reduction of lesions in foot ulcers of diabetic patients (Carvalho et al., 2016). Interestingly, *C. officinalis* has been considered as an alternative resource by national health surveillance agencies such as the one in Brazil.

### *Achillea* Genus

*Achillea* genus has been widely used in the traditional medicine as a source of healing products (Mohammadhosseini et al., 2017; Jaric et al., 2018). *Achillea millefolium* L. is the most studied species among others. *A. millefolium* is a herb, commonly known as yarrow, which is indigenous to the Northern Hemisphere of Europe and Asia, and it has been popularly used for over 3,000 years (Ali et al., 2017). Its pharmacological properties include anti-inflammatory, antioxidant, antifungal, and healing actions (Karamenderes and Apaydin, 2003; Fierascu et al., 2015), which have been attributed to several chemical constituents such as sesquiterpenes and phenolic compounds (Fierascu et al., 2015).

An *in vitro* study, carried out in human skin fibroblasts, showed that the hydroalcoholic extract from the aerial parts of *A. millefolium* induces cell proliferation (Ghobadian et al., 2015). More recently, oil extracts from aerial parts of *A. millefolium* were shown to reduce skin irritation in healthy individuals. Two approaches were applied to obtain the extracts: (i) the aerial parts of *A. millefolium* were macerated with ethanol, followed by olive oil (E1) or sunflower oil (E2) and (ii) the maceration of plant material occurred only in the presence of olive oil (E3) or sunflower oil (E4). This double-blind study enrolled 23 volunteers who had 8% sodium lauryl sulfate applied to their skin to cause irritation. After 24 h, these subjects received a topical application of the oil extracts for 7 days. All oil formulations were able to stabilize the skin pH and to increase hydration while reducing erythema. However, E1 and E2 exhibited the highest anti-inflammatory action, whereas E3 and E4 promoted highest levels of skin hydration. The presence of compounds with reported anti-inflammatory actions in both E1 and E2 (luteolin, apigenin and their glycosides, caffeic, and chlorogenic acids as well as chlorophyll derivatives) may explain these results (Tadic et al., 2017).

Evidences have also suggested a healing potential of *Achillea asiatica* Serg. (synonym of *A. millefolium* var. *manshurica* Kitam), popularly known as Mongolian yarrow. *In vitro* incubation of the ethanolic extract from the aerial parts of this plant with Hs68 fibroblasts triggered the production of collagen by these cells; this involved the activation of transforming growth factor-β-mediated pathways. The same extract also enhanced the differentiation and motility of keratinocytes through the upregulation of β-catenin, Akt, and keratinocyte differentiation markers. Compounds such as chlorogenic acid, apigenin-7-*O*-glucoside, and schaftoside were identified and associated with the healing effects of *A. asiatica* ethanolic extract (Dorjsembe et al., 2017). A comparative analysis of the *in vitro* healing potential of extracts obtained from *A. coarctata*, *A. kotschyi*, and *A. lycaonica* was performed in cultured NIH-3T3 fibroblasts. *A. kotschyi* extract was the most effective, presenting chlorogenic acid, hyperoside, apigenin, hesperidin, rutin, kaempferol, and luteolin in its composition (Jain et al., 2015).

### *Pluchea* Genus

Plants from the *Pluchea* genus have been used as healing agents by different communities (Gridling et al., 2009; Schmidt et al., 2009; Ab Rahman et al., 2014). *Pluchea indica* (L.) Less. healing actions have been attributed to its antioxidant and anti-inflammatory properties (Buapool et al., 2013). These evidences have been further supported by recent studies showing that nanoparticles containing *P. indica* leaf ethanolic extract increase the migration of oral mucosal cells *in vitro*. This preparation presented characteristics (size, charge, polydispersity index, increased colloidal stability) that support its use as an oral spray (Buranasukhon et al., 2017). Furthermore, the size of *Leishmania amazonensis*–induced cutaneous lesions in BALB/c mice was found to be reduced by the intralesional treatment with an essential oil obtained from the leaves of *Pluchea carolinensis* (Jacq.) D.Don. The main component of this essential oil was selin-11-en-4α-ol (Garcia et al., 2017).

### *Artemisia princeps* Pamp. and Isolated Compounds

**FIGURE 3:**
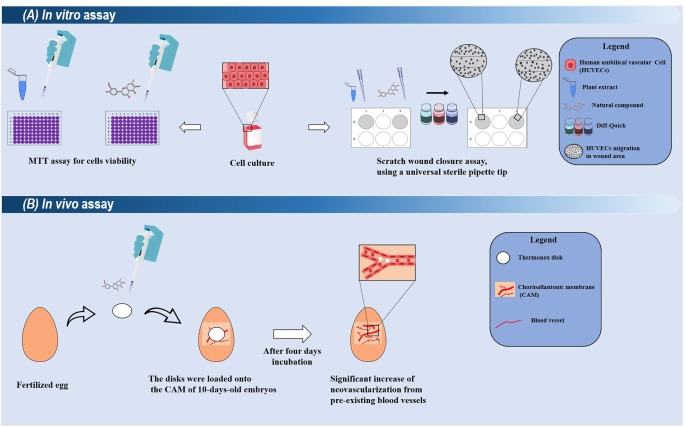
Summarized representations of *in vitro* and *in vivo* techniques used for evaluating the healing potentials of plant-derived compounds. **(A)** In *in vitro* assays, the cytotoxic and proliferative effects of a compound/extract can be evaluated toward different cell cultures (such as human umbilical vascular cells). Their proliferative effects can be analyzed through the scratch wound closure assay. Compounds with promising results can be selected for *ex vivo* and *in vivo* models. **(B)** A simple *in vivo* model using chick Chorioallantoic membrane (CAM). In this assay, the damaged CAM is treated with the selected compound (applied into a disk), and after few days, the neovascularization is measured.

Another plant with the ethnopharmacological relevance is *Artemisia princeps* Pamp., which is traditionally used to treat inflammatory-related diseases and had its properties scientifically proven in various *in vitro* and *in vivo* models (Min et al., 2009; Chen et al., 2016). Jaceosidin is extracted from this plant, which has also been identified as the main constituent of other plants from the *Artemisia* genus such as *A. argyi* with ethnomedicinal use as a healing agent (Li et al., 2018). It has the ability to inhibit the production of pro-inflammatory mediators such as TNF-α, IL-1β, and PGE_2_ (Min et al., 2009). *In vitro*, this flavonoid induces the proliferation, migration, and differentiation of human umbilical vascular endothelial cells (**Figure 3**) (Lee et al., 2014). It also stimulates the formation of microvessels in rat aortic tissue, and this effect has been associated with the activation of VEGFR2/FAK/PI3K/AKT/NF-κB signaling pathways (Lee et al., 2014). Overall, all these studies suggest Jaceosidin as an interesting pro-angiogenic compound.

Isosecotanapartholide, isolated from *A. princeps*, has also exhibited *in vitro* proliferative properties. Isosecotanapartholide (and the extract from *A. princeps*) inhibited the production of IL-33 by human keratinocytes (HaCaT), and this was associated with reduced levels of signaling molecules such as signal transducer and activator of transcription-1 (STAT-1), thymus and activation-regulated chemokine (TARC/CCL17), and adhesion molecule-1 (Ali et al., 2017).

### *Ageratina pichinchensis* (Kunth) R.M. King and H. Rob.

*Ageratina pichinchensis* (Kunth) R.M. King and H. Rob. is a plant with ethnopharmacological relevance in Mexico, and its several pharmacological activities have been confirmed in murine models and clinical trials *in vivo*, such as onychomycosis (Romero-Cerecero et al., 2009), interdigital tinea pedis (Romero-Cerecero et al., 2012b), stomatitis (Romero-Cerecero et al., 2015b), and vulvovaginal candidiasis (Romero-Cerecero et al., 2017). Despite its use for wound healing, the first study showed that the daily topical application of an aqueous extract from the aerial parts of *A. pichinchensis* heals wounds in rats without inducing skin irritation (Romero-Cerecero et al., 2011). Based on these results, a bio-guided purification revealed that 7-*O*-(β-D-glucopyranosyl)-galactin is the major compound associated with the effects of *A. pichinchensis* in cell proliferation (Romero-Cerecero et al., 2013). Later, two extracts (aqueous and hexane) with standardized concentrations of 7-*O*-(β-D-glucopyranosyl)-galactin were shown to promote the healing of skin lesions in rats with streptozotocin-induced diabetes (Romero-Cerecero et al., 2014).

The healing properties of this plant were also assessed in human clinical trials. For instance, the effectiveness of a standardized extract of *A. pichinchensis* was proved to heal chronic venous leg ulcers (Romero-Cerecero et al., 2012a). In another study, a cream containing an extract of *A. pichinchensis* was topically used by diabetic patients with foot ulcer; the results showed this treatment decreases healing time and lesion size although no significant differences were observed. The authors attributed this fact to the sample size, but they concluded that a large clinical trial could prove the action of *A. pichinchensis* in this type of wound (Romero-Cerecero et al., 2015a).

### *Achyrocline alata* (Kunth) DC. and *Achyrocline satureioides* (Lam.) DC.

Plants from the *Achyrocline* genus play an important role in traditional medicine and are commonly found in Latin American countries (Retta et al., 2012; Alerico et al., 2015; Bolson et al., 2015). Ethnobotanical surveys performed in the Brazilian state of Rio Grande do Sul indicated that *Achyrocline satureioides* (Lam.) DC. is widely used for healing. It was shown that the ethanolic extracts from the aerial parts of this plant induce the proliferation of HaCaT keratinocytes (Alerico et al., 2015). The healing activity of an essential oil of *A. satureioides* inflorescences incorporated into hydroxyethyl cellulose films was also demonstrated in Wistar rats (Yamane et al., 2016).

A recent study evaluated the use of the extracts from inflorescences of *Achyrocline alata* (Kunth) DC. and *A. satureioides* for the repair of cutaneous wounds in mice. Both extracts showed positive results, but only *A. alata* accelerated wound closure, presenting a higher probability of healing in a shorter time of treatment. The authors attributed this effect to higher concentrations of phenolic compounds in *A. alata.* Moreover, it was possible to observe that animals treated with *A. alata* extract present less mast cells at the site of inflammation, better re-epithelization and granulation of the injured tissue, and reduction of the initial inflammatory reaction (Pereira et al., 2017).

### *Acmella oleracea* (L.) Spreng.

*Acmella oleracea* (L.) Spreng. (jambu) is a native plant from Brazil that is used to treat skin and gastrointestinal disorders and also as a female aphrodisiac (Neamsuvan and Bunmee, 2016; Neamsuvan and Ruangrit, 2017; Da Rocha et al., 2018). A polysaccharide extracted from *A. oleracea*, named rhamnogalacturonan, was found to inhibit ethanol-induced gastric ulcers in rats (Nascimento et al., 2013). This effect was better elucidated later, as this compound was shown to protect against both acute (intraperitoneal treatment) and chronic lesions (oral administration) induced by ethanol (Maria-Ferreira et al., 2014). Rhamnogalacturonan also enhanced the gastric cell proliferation and mucus content while decreasing inflammation and oxidative stress in the stomach (Maria-Ferreira et al., 2014).

Another study reported the development of hydroxyethyl cellulose (HCE) films containing an ethanolic extract from the aerial parts of *A. oleracea* and an essential oil obtained from the inflorescences of *Achyrocline satureioides*. The HCE films containing these two plant materials demonstrated wound healing activity in Wistar rats, an effect that was associated with increased levels of collagen deposition in wounds. α-Humulene and spilanthol were detected in the essential oil of *A. satureioides* and the extract of *A. oleracea*, respectively (Yamane et al., 2016).

### *Artemisia* Plants

The genus *Artemisia* plays an important role in the traditional medicine (Shenkman and Krivenkov, 1986; Kadioglu et al., 2017; Ota and Ulrih, 2017) and in the development of anti-inflammatory and anticancer drugs (Kadioglu et al., 2017; Coricello et al., 2018; Konstat-Korzenny et al., 2018). The pharmacological potentials of these plants have also been evaluated in healing models. For example, the extract from *Artemisia asiatica* (Pamp.) Nakai ex Kitam was efficient against gastric injuries induced by ethanol (Park et al., 2008), while *Artemisia argyi* H.Lév. and Vaniot healed oral ulcers in rats (Yin et al., 2017). Another study showed that the essential oil from *Artemisia montana* (Nakai) Pamp improves the proliferation of human keratinocytes and enhances their capacity to produce type IV collagen. These effects were associated with the phosphorylation of Akt and ERK 1/2. *In vivo* assays showed that the essential oil from *A. montana* promotes the healing of rats with dorsal wounds (Yoon et al., 2014). The aqueous extract from *Artemisia campestris* L. also reduced the number of inflammatory cells in the wounded area and presented a positive effect in the progress of wound healing (Ghlissi et al., 2016).

## Conclusion

This review described the aspects involved in the healing properties of some Asteraceae plants. In fact, several plants from this family have ethnopharmacological relevance for the treatment of wounds due to their direct effects on healing and in some cases due to their anti-inflammatory actions. The discussed studies provided the scientific basis for the ethnopharmacological usage of these plants, since different products derived from them (isolated compounds, oils, and extracts) are effective in the models of healing *in vitro* and *in vivo*. Silibinin (from *S. marianum*) and jaceosidin (from *A. princeps*) were identified as promising compounds for the development of healing agents. Furthermore, the results obtained in clinical trials with *A. pichinchensis* and *C. officinalis* are exciting and highlight their importance for the treatment of wounds. These evidences suggest that Asteraceae plants are important sources for the development of new efficient drugs for healing.

## Author Contributions

AC, RD, MS, CF, AZ, MG, EF, and LdS contributed to conception and design and critically revised the manuscript. All authors gave final approval and agree to be accountable for all aspects of the work.

## Conflict of Interest Statement

The authors declare that the research was conducted in the absence of any commercial or financial relationships that could be construed as a potential conflict of interest.
